# Cellular senescence and acute kidney injury

**DOI:** 10.1007/s00467-022-05532-2

**Published:** 2022-03-26

**Authors:** Xiaoxi Lin, Heng Jin, Yanfen Chai, Songtao Shou

**Affiliations:** Department of Emergency Medicine, Tianjin Medical University General Hospital, Tianjin, China

**Keywords:** Acute kidney injury, Senescence, Chronic kidney disease, Senescence-associated secretory phenotype

## Abstract

Acute kidney injury (AKI) is a common clinical complication characterized by a sudden deterioration of the kidney’s excretory function, which normally occurs secondary to another serious illness. AKI is an important risk factor for chronic kidney disease (CKD) occurrence and progression to kidney failure. It is, therefore, crucial to block the development of AKI as early as possible. To date, existing animal studies have shown that senescence occurs in the early stage of AKI and is extremely critical to prognosis. Cellular senescence is an irreversible process of cell cycle arrest that is accompanied by alterations at the transcriptional, metabolic, and secretory levels along with modified cellular morphology and chromatin organization. Acute cellular senescence tends to play an active role, whereas chronic senescence plays a dominant role in the progression of AKI to CKD. The occurrence of chronic senescence is inseparable from senescence-associated secretory phenotype (SASP) and senescence-related pathways. SASP acts on normal cells to amplify the senescence signal through senescence-related pathways. Senescence can be improved by initiating reprogramming, which plays a crucial role in blocking the progression of AKI to CKD. This review integrates the existing studies on senescence in AKI from several aspects to find meaningful research directions to improve the prognosis of AKI and prevent the progression of CKD.

## Introduction

Acute kidney injury (AKI) places a great economic burden on high-income or low-to-middle income countries, with extremely high morbidity and mortality and no specific and efficient treatment measures other than dialysis to relieve symptoms. Although AKI was previously often deemed as a self-limiting disease, it is now considered that acute decline in kidney function is associated with long-term consequences, including progression to chronic kidney disease (CKD), kidney failure, sustained functional impairment, and death [1]. After mild injury, the kidney may return to a structural and functional state that is indistinguishable from normal. This is due to the strong compensatory ability of mature kidney cells, which can quickly re-enter the cell cycle from a resting state within 24 h after injury. If the kidney is severely impaired or damaged, it will cause a variety of pathological changes and interstitial fibrosis. In one study, the survivors of severe AKI had a worse health-related quality of life (HRQOL) as compared to the general population, and both physical and mental components of these survivors were affected [2]. Given these undesirable outcomes, effective measures are required to reduce kidney damage, delay kidney function deterioration, and improve kidney function recovery. To resolve this issue, it is essential to elucidate the mechanisms of AKI and eliminate their components. In recent years, senescence has increasingly attracted the attention of researchers because of its role in the development and progression of AKI.

To date, current research has demonstrated that senescence is a state of the stable arrest of the cell cycle and/or DNA damage, which is accompanied by alterations at the transcriptional, metabolic, and secretory levels along with modified cellular morphology and chromatin organization [3, 4]. Other characteristics of senescent cell cycle arrest include defects in ribosomal biosynthesis and de-inhibition of retrotransposons [5, 6]. Senescence is a regulated signal transduction process, wherein specific senescence-inducing signals contribute to diverse senescent consequences [7–10]. In a previous study on different kidney injury models, significant cellular senescence was observed 3 days after injury [11]. Several studies have also reported the relationship between kidney fibrosis and cellular senescence [12]. When AKI occurs, both the cortex and medulla of kidney tissue can be senescent, including renal tubular epithelial cells (TECs), podocytes, vascular smooth muscle cells, endothelial cells, and mesenchymal cells, among which renal TECs are the most common cells that undergo senescence [13]. Tubular cell senescence is an early central mechanism that leads to further accumulation of senescent cells after kidney injury and causes the progression of kidney damage [11]. A high amount of senescence-associated secretory phenotype (SASP) can be detected in both the acute phase of AKI and the CKD phase. Does senescence play a positive or negative role in AKI and CKD? What are the specific mechanisms involved? Are there any targeted measures for treatment to reduce the burden of AKI and CKD? This review summarizes the emerging knowledge on senescence underlying both AKI and CKD and the therapeutic opportunities they present.

## Senescence-associated secretory phenotype

Although the cell cycle of senescent cells is in a state of stagnation, the cells still maintain their metabolic activity and release a special secretory group that can affect neighboring cells and ultimately affect tissue function. This phenotype is defined as SASP that includes pro-fibrosis and pro-inflammatory factors such as interleukin-1β (IL-1β), IL-6, IL-8, transforming growth factor-β1 (TGF-β1), monocyte chemoattractant protein 1 (MCP-1), plasminogen activator inhibitor 1 (PAI-1), and cellular communication network factor 2 (CCN2), which function through a paracrine and autocrine manner [14, 15]. Many studies have shown that the intense inflammatory process involved in the innate and adaptive immune response can cause initial kidney damage and mediate long-term structural changes, including interstitial fibrosis or repair. The balance between pro-inflammatory factors and anti-inflammatory factors is extremely important for tissue repair [14, 16, 17]. In every specific senescent cell, SASP involves not a single component but a combination of multiple components and has great variability. The composition of SASP depends on the affected cell type, the nature of the stressor, and the stage of senescence; hence, its composition changes dynamically [18]. It is speculated whether the terminal outcome of senescence depends on the dynamic development of SASP. SASP affects neighboring cells through the autocrine positive feedback loop and paracrine signaling, which amplifies the senescence signal and leads to a continuous progression of senescence [19]. An increase in SASP has been observed in elderly mice with AKI, including TGF-β1, IL-6, and CCN2 [15]. The kidney ischemia–reperfusion model shows significant TNF secretion within the first 24 h [20]. SASP secreted by senescent cells acts on the surrounding healthy cells, which further aggravates cell senescence and fibrosis, leading to CKD [21]. Many studies have shown that the intense inflammatory process involved in the innate and adaptive immune response can cause initial kidney damage and mediate long-term structural changes, including interstitial fibrosis or repair. SASP also causes activation, attraction, and secretion of immune cells in the process of acute senescence to eliminate senescent cells, which is called “immunity supervision” [22]. Experiments have shown that SASP can eliminate senescent cells accumulated after chemotherapy to prevent tumor recurrence [19]. SASP is harmful in the long term, but in the short term can also play a beneficial role. For example, vascular endothelial growth factor (VEGF) and fibroblast growth factor 2 can promote tissue repair following kidney damage [23]. However, it seems that its harmful effects outweigh the beneficial effects. Studies have shown that SASP secreted by senescent fibroblasts promotes the proliferation and metastasis of pre-cancerous epithelial cells and increases tumor angiogenesis to promote cancer progression [24]. It is also thought that IL-6, TNF-α, and MCP-1 can recruit some inflammatory cells to promote damage repair, but this might also lead to continuous expression of SASP, resulting in undesirable outcomes [12]. Therefore, the role of SASP in AKI and CKD should be analyzed in a more specific scenario. The dual role of SASP may correspond to the acute senescence stage and the chronic senescence stage. Compilation of SASP components, beneficial effects of SASP, and senescence-related pathways are shown in Table 1.

**Table 1:** Compilation of SASP components, beneficial effects of SASP, and senescence-related pathways

Composition of SASP	Inflammatory cytokines, immune modulators, growth factors, proteases, exosomes, ectosomes … (IL-1β, IL-6, IL-8, TGF-β1, TGF-β3, IL-12, IL-10, TNF…)
Benefits of SASP	SASP can help signal immune cells to achieve senescent cell clearance, and specific SASP factors secreted by senescent cells attract and activate different components of the innate and adaptive immune systems; SASP recruit macrophages to remove cancer cells; SASP factors can maintain senescent cells in a growth-arrested senescent state, thereby preventing carcinogenesis; SASP can promote wound healing; SASP can promote tissue remodeling…
Senescence pathways	DNA damage response → ATR → p53 → p21 → Cyclin → pRB → SAHF → HP1; DNA damage response → ATM → p53 → p21 → Cyclin → pRB → cell cycle arrest; Oncogenic RAS → ROS → ERK → ETS → p16 → Cyclin → pRB → cell cycle arrest; Oncogenic RAS → ROS → ERK → P38/MAPK → p53 → p21 → Cyclin → pRB → cell cycle arrest; mTOR → p53 → p21 → CDK2 → RB1…

## Acute cellular senescence

With the advancement in research, several lines of evidence show that senescence has unique manifestations at specific stages of cells, which differentiate acute senescence from chronic senescence. Acute cellular senescence is a beneficial and specific physiological process, which has the characteristics of clear senescence trigger, short-term senescence signal, and rapid senescence cell clearance, and the whole process is strictly controlled. Acute senescence causes the cell cycle to be temporarily blocked, which helps cells to avoid uncontrolled mitosis and provides more time for DNA repair [25, 26]. Senescent cells were discovered in the process of the development of mouse embryos, and they were shown to participate in the reshaping of the mesonephros, the endolymphatic sac, the apical ectodermal ridge, and the neural roof plate [10, 27]. Senescence is important for maintaining tissue homeostasis after injury. Senescent cells play a positive role in damage repair, which can promote skin wound healing [28, 29] and repair of the damaged liver [30]. Senescent cells also prevent tumorigenesis and transformation by inducing cell cycle arrest [31, 32]. In the abovementioned cases, the SASP produced by senescent cells leads to a common pathway to clear senescent cells, limit fibrosis, and prevent undesirable outcomes, which is called “immune surveillance” [33, 34]. In an earlier study in different kidney injury models, significant cellular senescence was observed 3 days after injury, which manifested as an increase in the activity of the enzyme senescence–associated β-galactosidase (SA-β-Gal) and a reduction in the protein abundance of Lamin B1 (LAMNB1) nuclear membrane [11]. In the early stages after the onset of AKI, the cell cycle is blocked by specific inhibitors to provide time for DNA repair and avoid excessive progression to apoptosis in the damaged renal tubular cells [21]. The use of cyclin-dependent kinase (CDK) inhibitors to arrest the cell cycle of damaged renal TECs ensures more time to repair damaged DNA and reduces cell senescence and CKD [35]. The course of acute cellular senescence is short and programmed. In general, the strong compensatory ability of the kidney enables it to respond to injury through adaptive repair. After the acute phase, the senescent cells are eliminated and no longer secrete SASP, thereby leaving no effect on long-term kidney function. However, in the studies conducted to date, the effects of senescence on AKI have been negative. Even if the injury has ceased, kidney inflammation and fibrosis continue to progress. Maladaptive TECs after AKI are considered as a leading cause of renal fibrosis post-AKI [36]. Repeated or extremely severe injuries within a short period of time lead to renal maladaptive repair, persistent inflammation, and cell cycle arrest of renal TECs at the G2/M phase. The cell cycle arrest at the G2/M phase promotes the secretion of inflammatory factors and pro-fibrotic factors to aggravate the damage and form a vicious circle [37]. This phenomenon is called “chronic cellular senescence.”

## Chronic cellular senescence

Different from acute senescence, chronic senescence does not have a specific program, but it is a random process. Multiple and persistent stresses act on tissues and organs. Senescent cells cannot be effectively eliminated. An increasing number of senescent cells accumulate and induce more severe senescence by autocrine and/or paracrine secretion of SASP, which eventually lead to organ dysfunction [12]. This aspect is a major difference between acute senescence and chronic senescence. Studies have also shown that the accumulation and continuous secretion of SASP by senescent cells cause epithelial–mesenchymal transition (EMT) in fat and heart and kidney tissues and shortened lifespan [38–40]. Age-related conditions, tissue degradation, and body aging are related to chronic senescence. In addition, another difference between acute and chronic senescence is the cell cycle arrest time. A short cell cycle arrest may not cause an irreversible outcome, while a permanent cell cycle arrest may trigger a series of pathological changes [26]. If immune clearance is impaired with age, chronic senescence will evolve from acute senescence, leading to long-term stagnation and possible changes in SASP [41]. An epithelial injury is a core event of CKD. A previous study showed that after AKI, the injured epithelial cells can de-differentiate and re-enter the cell cycle to proliferate and return to normal function; this is called adaptive repair [42]. It may be more accurately called “reprogramming”. Kidney injury molecule 1 (KIM-1) is markedly upregulated in the proximal tubule after injury. Persistent expression of KIM-1 is maladaptive and adequate to contribute to leukocyte infiltration, tubular atrophy, CKD, and ultimately, kidney failure [43]. Slight insult to the kidney can be offset by adaptive repair. Repeated and severe insult in a short period of time will cause renal TECs to undergo cell cycle arrest; produce inflammatory factors and pro-fibrotic factors, and cause cell loss, tissue damage, and kidney function loss [44]. CKD shows the characteristics of senescence: 28 days after kidney injury, the activity of SA-β-Gal in renal TECs increased, LAMNB1 decreased, and the expression of pro-inflammatory cytokines IL-1α, IL-1β, IL-6, TNF-α, and MCP-1 increased [11]. Fibrosis is a representative feature of CKD. The maladaptive repair that leads to CKD is characterized by persistent inflammation and fibroblasts. To sum up, the conversion of AKI to CKD seems to correspond to the transformation of acute cellular senescence to chronic cellular senescence. With regard to different outcomes of AKI, in addition to adaptive repair and CKD, cellular reprogramming also plays a key role.

## Cellular reprogramming

**Fig. 1:**
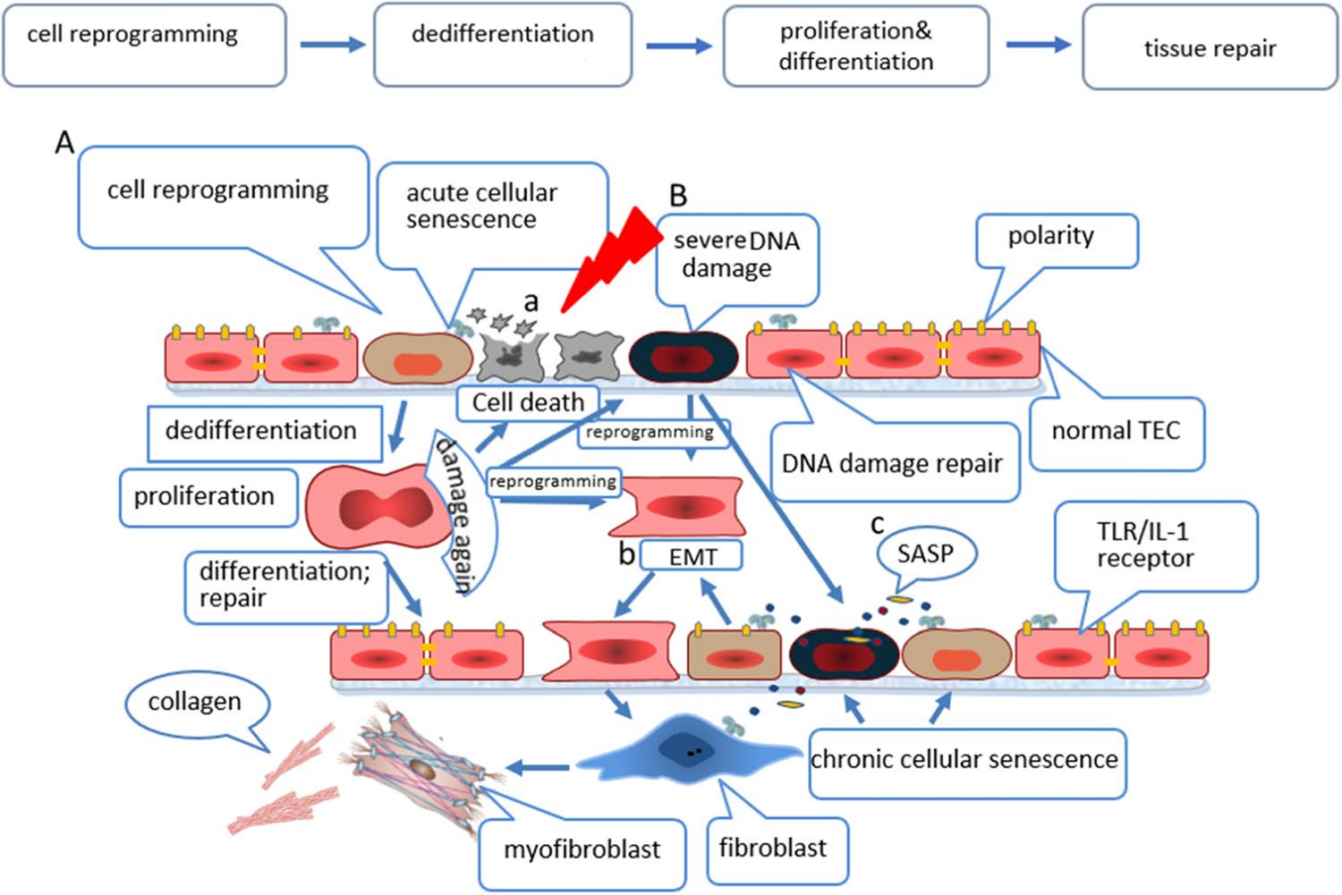
Different outcomes of renal tubular cells after injuries: A after a mild injury, the cells can be completely repaired through reprogramming (adaptive repair); B after a repeated or severe injury: a, cell necrosis or apoptosis; b, EMT contributes to fibrosis and collagen deposition after reprogramming; c, SASP secretion evolves into chronic cellular senescence

It is known that cellular reprogramming is the process of reverting differentiated cells to the pluripotent state, and the pluripotent state can be differentiated into various functional cells [45, 46]. There are diverse modes of reprogramming. Cells can first return to a pluripotent state and then differentiate into desired lineages [47]. Alternatively, one cell type can directly convert into another cell lineage by expressing specific factors [48, 49]. Takahasi and Yamanaka showed for the first time that mouse somatic cells can be reprogrammed into a pluripotent state by expressing four transcription factors (Oct4, Sox2, Klf4, and c-Myc) [46]. Reprogramming initially triggers a stress response characteristic of senescence. Banito et al. infected IMR90 cells with four reprogramming factors (Oct4, Sox2, Klf4, and c-Myc). Each of the four factors reduced cell growth and showed SA-β-Gal activity and senescence-related heterochromatic foci. The expression of reprogramming factors in human fibroblasts (BJ) or mouse embryonic fibroblasts (MEF) also leads to growth arrest with senescence characteristics. The authors also observed that reprogramming factors caused DNA damage response and expression of tumor suppressor factors p16^Ink4a^, p53, and p21^Cip1^, indicating that there is an inherent relationship between senescence and reprogramming. The authors proposed that inhibiting senescence can increase the efficiency of reprogramming. Knockdown of p16^Ink4a^/p19^Arf^, p21^Cip1^, or p53 expression by shRNAs or experiments conducted in strains with a knockout of p53 or p21 has shown that the efficiency of reprogramming increases after senescence effectors are exhausted [50]. Early studies have demonstrated that cell senescence in the process of AKI proliferation and repair is not an irreversible “on/off” process, and its cell phenotype changes dynamically. In the process of tissue damage and repair, cell senescence and secreted SASP after injury are important factors leading to cell reprogramming [51]. This explains why only a part of the cells in the injury site can be reprogrammed, which shows a certain degree of restriction on reprogramming. The p53/p21^Cip1^ pathway is involved in the expression of response to reprogramming factors at different levels. Data obtained from the individual expression of reprogramming factors indicate that the activation of p21^Cip1^ appears to be the key endpoint for the convergence of different signals [50]. IL-6 secreted by senescent cells stimulates the surrounding normal cells to produce four reprogramming factors (Oct4, Sox2, Klf4, and c-Myc) through paracrine signaling to stimulate reprogramming [52]. Senescence inhibitors reduce senescence by inducing mitochondrial reprogramming in animal experiments [53]. We speculate that the degree of SASP secretion affects the efficiency of reprogramming and further affects the outcome of the tissue after injury. After a mild injury, cells secrete a small amount of SASP without affecting the normal progression of reprogramming to complete repair. This process may correspond to adaptive repair during acute senescence. In contrast, severe injury hinders reprogramming, the damage cannot be fully repaired, and SASP is continuously secreted, thereby causing EMT or chronic cell senescence, and the final outcome is CKD or kidney failure. The mechanisms for the transition from AKI to CKD include endothelial cell damage, cell cycle arrest, epigenetic changes, sparse blood vessels, interstitial fibrosis, and cellular metabolic reprogramming [54, 55]. Fatty acid oxidation (FAO) is the main energy supply pathway for the metabolism of proximal renal tubular cells (PTCs). During AKI, FAO is abolished, and PTCs undergo metabolic reprogramming, which increases the risk of AKI to CKD conversion [56]. EMT is a mechanism of reprogramming, and EMT may be involved in the pathogenesis of renal fibrosis [57]. EMT transforms epithelial cells into myofibroblasts; these myofibroblasts proliferate and produce a fibrotic matrix [58]. A prostaglandin E-2 receptor 4 (EP4) antagonist can inhibit the de-differentiation of renal tubular cells, which targets EMT [59]. Conversely, cells in acute senescence can undergo cellular reprogramming to de-differentiate, proliferate, and differentiate to achieve tissue regeneration and repair in AKI. There are currently two controversial claims that the repair of cells after AKI is achieved by de-differentiation of differentiated cells or by direct differentiation of progenitor cells. Endothelial progenitor cells of rats with renal ischemia–reperfusion injury secrete microvesicles through the paracrine pathway, which activates an angiogenic program to reverse AKI, glomerulosclerosis, and tubular interstitial fibrosis to prevent the progression of chronic kidney injury [60, 61]. The proliferation and repair of renal TECs after AKI are achieved by the dedifferentiation of differentiated cells and their re-entry into the cell cycle [62]. After renal ischemia–reperfusion injury, the surviving renal TECs will de-differentiate and proliferate, and eventually replace the irreversibly damaged renal TECs, thus restoring the integrity of renal tubules [63]. Epithelial cell proliferation helps to restore cell necrosis and apoptosis. Proliferation capacity is the main feature of PTCs. The ability of tubular cells to transform into different cell types is called cell plasticity. The plasticity of renal tubular cells allows them to resist acute injury and proliferate (this process is also called reprogramming) [64]. On the basis of the above brief description, reprogramming plays a crucial role in the outcome of AKI. On the one hand, it promotes the repair of damage caused by AKI (this may correspond to the efficient performance of acute cellular senescence), and on the other hand, it develops into CKD or kidney failure through EMT in a poor environment. Different outcomes of renal tubular cells after injury are shown in Fig. 1.

## Senescence-related signaling pathways

At present, known senescence-inducing factors include oxidative stress, cytotoxicity, mitochondrial dysfunction, repeated cell division, and telomere shortening, and the latter two are called “replicative senescence” [3, 65]. Most of the inducing factors initiate cell cycle arrest through the cyclin-dependent kinase (CDK) inhibitors p16^Ink4a^ and/or p21^Cip1^. The two most core signaling pathways are the p16^Ink4a^/Rb pathway and the p19^Arf^/p53/p21^Cip1^ pathway, which interact but independently adjust the process of the cell cycle [41, 66, 67]. p16^Ink4a^ and p21^Cip1^ belong to CDK inhibitors, where p16^Ink4a^ is referred to as cyclin-dependent kinase inhibitor 2A (CDKN2A) and p21^Cip1^ is known as cyclin-dependent kinase inhibitor 1A (CDKN1A); cyclins, CDKs, and CDK inhibitors are proteins that regulate the cell cycle. p16^Ink4a^ plays a key role in downregulating CDK4 and CDK6 during cell senescence. p21 induces G1 phase block to escape DNA damage during AKI, while p21 knockout mouse with AKI showed high mortality [68]. p16^Ink4a^ and p21^Cip1^ are the core hubs of senescence. There are many confirmed pathways and still others waiting to be developed. For example, rapamycin alleviates senescence induced by Dox or H_2_O_2_ through the mTORC1/p70S6K pathway in mesenchymal stromal cells, which is accompanied by a decrease in p21, p53, and p16 [69]. Reactive oxygen species (ROS) activate the c-Jun NH-2 terminal kinase (JNK) pathway, which induces FOXO transcription factors that cause DNA damage and cell cycle arrest [70]. SASP components can be controlled by Rel/NF-κB transcription factors. Ras activation triggers many downstream signaling pathways, including the Raf/MEK/MAPK pathway, the phosphoinositide 3-kinase (PI3K) pathway, and normal cell proliferation induced by increased formation of ROS, leading to cell cycle arrest and senescence [71]. There are specific descriptions regarding the senescence pathways in different senescence models. Next, we briefly describe the senescence pathways in AKI and CKD. AKI is usually accompanied by the presence of DNA damage response (DDR), which leads to ataxia telangiectasia mutation (ATM) and/or ataxia telangiectasia and activation of Rad3-related (ATR) proteins; both these proteins are members of the phosphatidylinositol 3-kinase family that phosphorylates several downstream targets, including p53 and checkpoint kinase 2 (CHK2), and subsequently, produce p21^Waf1/Cip1^. p21^Waf1/Cip1^ is a cell cycle inhibitor that can be used to stop the cell cycle of renal TECs in the G1 or G2/M phase [72]. In various AKI models (cisplatin, ischemia–reperfusion injury, and unilateral ureter obstruction), Rad3-related ATR activation after AKI protects against maladaptive tubular repair, resulting in less fibrosis. ATR arrests the cell cycle of proximal tubules at the G2/M phase by activation of DDR signaling [73]. Mitochondrial dysfunction occurs in renal TECs in ischemic AKI, which produces a large amount of ROS to activate the p53/p21^Cip1^ pathway [74]. A high amount of ROS molecules can cause significant modification of lipids, DNA, and proteins [75]. In addition, ROS cause DNA damage and cell cycle arrest. Telomere shortening of renal TECs after AKI has been confirmed to aggravate the senescence process. The senescence-related protein Klotho mainly exists in renal proximal tubule epithelial cells (PTEs). Mice lacking Klotho show various senescence-related features. Klotho has been used as an important target for anti-senescence treatment. Experiments have shown that AKI caused by epigenetic stress activates the p16^Ink4a^ pathway [76]. In the AKI model, p16^Ink4a^, p21^Cip1^, and TGF-β were overexpressed within 2 days of injury, and high levels of β-gal and p16^Ink4a^ were maintained after ischemic injury [76]. The G2/M block of renal TECs is an important driving force for maladaptive repair and progressive CKD after AKI [77]. Overexpression of TAZ in HK-2 cells can cause G2/M cell cycle arrest and de-differentiation and promote the production of pro-fibrotic cytokines, which indicates that the Hippo/YAP/TAZ signaling axis is involved in the senescence of renal TECs [78]. It is generally believed that the cells that stagnate after a minor injury will enter the cell proliferation stage after 1–5 days in the G2/M phase and will not produce fibrosis and leave scars, which shows the characteristic of “self-limiting.” Another research study suggested that senescence occurs in the early 2–3 days after acute injury, which is mediated by epithelial toll-like and interleukin 1 receptors (TLR, IL-1R) in a cell-independent process [11, 79]. A major injury causes the cell cycle to stagnate in the G2/M phase for a long time, thereby activating the c-Jun NH-2 terminal kinase (JNK) signal to produce fibrosis. During the period of cell cycle arrest, cells appear to be in a state of senescence. At this time, senescent cells may secrete SASP to trigger the senescence signaling pathway and then act on other senescent cells and surrounding cells to aggravate pathological changes and form a “vicious circle”. Among the SASP components, connective tissue growth factor and TGF-β can cause chronic inflammation, collagen deposition, and angiogenesis. In addition, a previous study demonstrated that eliminating cell senescence is beneficial to maintain kidney function, and the reduction of fibrosis by blocking tubular innate immunity through epithelial cell-specific deletion of Myd88 reduces the expression and secretion of SASP. Deletion of Myd88, a downstream effector of the TLR/IL-1R pathway upstream of NF-kB, reduced kidney damage and fibrosis [11, 79]. Pericytes are one of the main sources of myofibroblasts that form scars in CKD. Interestingly, genetic and pharmacological elimination of senescent cells partially prevents fibrosis but does not protect kidney function.

**Fig. 2:**
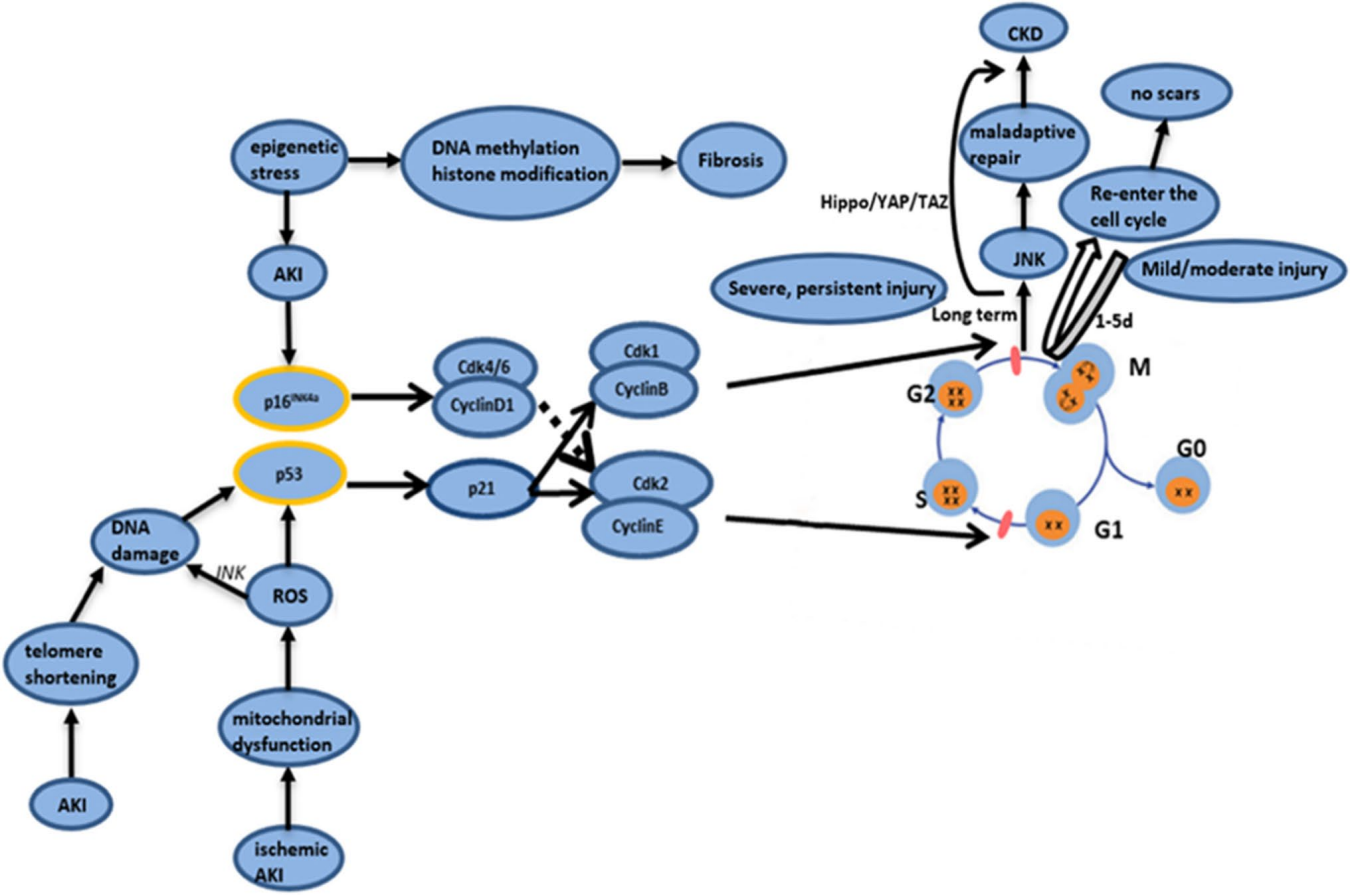
This figure shows some of the pathways through which stress induces cell cycle arrest after acute injury in the kidney. Telomere shortening and mitochondrial dysfunction cause DNA damage, and the activation of the p53/p21 pathway inhibits CDK2 and CDK1, leading to cell cycle arrest in the G0/G1 or G2/M phase. Other epigenetic stress activates the p16 pathway and inhibits CDK4/6, thereby blocking the cell cycle at the G0/G1 phase. After mild and moderate injuries, the cell cycle is temporarily arrested, and proliferation resumes without fibrosis. Severe and continuous injuries cause long-term cell cycle arrest through the induction of CKD by the JNK, Hippo/YAP/TAZ, and other pathways

To date, it is believed that the main determinant of the development of chronic cell senescence after AKI is the degree of damage. Chronic senescent cells produce maladaptive repair, which is characterized by interstitial fibrosis, renal tubular atrophy, and thinning of capillaries. Maladaptive repair hinders the complete recovery of kidney morphology and function, and it is probably the main reason for the progression of AKI into CKD [13]. A key feature of maladaptive repair after AKI is the increase in the number of fibroblasts, which leads to the deposition of collagen and other components of the fibrotic matrix in the kidney. The sources of fibroblasts include circulating fibroblasts, EMT, pericytes, and perivascular fibroblasts [22]. Risk factors for maladaptive repair include the type and duration of injury, the glomerular filtration rate before an injury, and age. Existing studies have shown that the Wnt signaling pathway plays a role in the senescence of renal TECs and fibrosis. Wnt9a significantly upregulates the levels of p16^Ink4a^, p53, and p21 related to senescence; induces senescent tubule cells to produce TGF-β1; and promotes the growth, proliferation, and activation of fibroblasts in normal rat kidneys. This includes the Wnt9a-TGF-β signaling pathway, which is an evolutionarily conserved pathway involved in organ development and tissue repair [22]. Klotho deficiency activates the Wnt/β-catenin signaling pathway, which further arrests cells in the G2/M stage and induces cell senescence in mice with ischemia–reperfusion-AKI [80]. On the other hand, recent literature shows that premature immune senescence accounts for CKD progression. Premature replicative senescence in young patients with CKD was found to be associated with poor naive T cell frequency and decreased thymic output [81]. Figure 2 shows some of the pathways through which stress induces cell cycle arrest after acute injury in the kidney.

## Biomarkers of senescence

The markers currently used to detect senescence include SA-β-gal, p16^Ink^, p21^Cip1^, TGF-β1, IL-6, and double-strand breaks (DSBs). Among them, β-gal is the most widely used marker, which reflects an improvement in the activity of the lysosome; however, the senescence function of cells lacking *GLB1* (the gene encoding lysosome β-gal) is not impaired, which limits its applicability as an independent marker for detecting senescence [18]. Presently, there is a lack of unique and specific markers of senescence in vivo and in vitro, and the presence or absence of one or more of the factors described above is not sufficient to confirm senescence [31]. The current methods to quantify tissue senescence include staining of cell cycle arrest markers, quantifying the release of aging-related proteins, and elimination of coexisting cell proliferation [82]. Previous studies have shown that lipofuscin coexists with SA-β-gal in senescent cells; however, a confirmation of this observation is lacking [18]. In addition, senescence can be detected by finding pathways that lead to cell cycle arrest. For example, the phosphorylation level of histone H2AX in senescence-related heterochromatin lesions, the increase of CDK inhibitors (p53, p21, and p16), and the elimination of p16-expressing cells can have a beneficial effect on the age-related decline. Lamin B1, a nuclear membrane component of senescent cells, is lost. Lamin B1 is downregulated by caspases in apoptotic cells, and the decrease in Lamin B1 in senescent cells is due to the decreased stability of Lamin B1 mRNA [83].

## Senolytic therapy

In a previous study, bone marrow mesenchymal stem cells transfected with Klotho were transplanted into mice with AKI, and kidney injury after renal ischemia–reperfusion was found to be significantly alleviated. Klotho inhibits the Wnt/β-catenin pathway in renal TECs [84]. Indoleamine 2,3-dioxygenase 1 (IDO) activates the general control nonderepressible-2 kinase (GCN2K) to facilitate DDR in ischemia–reperfusion-induced renal TECs. The IDO inhibitor 1-DL-methyl-tryptophane (1-MT) has been experimentally shown to inhibit senescence [85]. The B cell lymphoma (Bcl) 2/w/xL inhibitor ABT-263 reduced the number of senescent cells and restored the regenerative phenotype of the kidney after subsequent ischemic perfusion injury. However, the physiological network in the human body is complex. Eliminating a certain pathway could be positive for the treatment of senescence but may cause other adverse effects. Long-term use of Bcl-2 inhibitors causes thrombocytopenia [86]. LXA4 pretreatment significantly restores kidney function and improves the survival rate of rats after cecal ligation and puncture (CLP). LXA4 inhibits NF-kappa B (NF/kB)–mediated inflammation and the p53/p21 senescence pathway in a PPAR-γ-dependent manner to alleviate kidney inflammation and renal TEC senescence [87]. Nicotinamide mononucleotide (NMN), when administered in advance or during the recovery phase, can reduce senescence and fibrosis in H_2_O_2_ and hypoxia kidney injury, and ischemia–reperfusion-AKI mice [88]. Cell cycle inhibitors such as roscovitine, olomoucine, and purvalanol that inhibit cell cycle kinase CDK2 and PD 0,332,991 (a cell cycle inhibitor of cell cycle kinase CDK4/6) that target p21 can block the cell cycle at the G0/G1 phase [89, 90]. Fasudil has a protective effect on DOX-induced nephrotoxicity in mice and NRK-52E cells. It can prevent oxidative stress and DNA damage by inhibiting the RhoA/Rho kinase (ROCK) signaling pathway, inhibit cell apoptosis, and delay cell senescence [91]. In various animal models of AKI, metformin protects renal tubular cells from inflammation, apoptosis, ROS, internal endoplasmic reticulum (ER) stress, and EMT. In diabetic nephropathy (DKD), metformin also relieves podocyte loss, mesangial cell apoptosis, and renal tubular cell senescence through the AMPK-mediated signaling pathways. As a common route from CKD to kidney failure, metformin can improve renal fibrosis, which depends largely on AMPK activation [92]. In addition, a large number of drugs have been used to alleviate senescence in various animal experiments and clinical trials; however, more accurate evidence is required for safe application of these drugs to patients in the long term, and this could be an area for future study.

## Conclusion

After acute injury, the kidney can overcome the injury and restore its functionality in the right state. However, on many occasions, the injury may be severe. Hence, AKI has a high mortality rate and poor prognosis, and it therefore must be taken seriously. Cellular senescence and reprogramming intertwine and interact in the progression from AKI to CKD. It is essential to use senescence as a key entry point for the prevention and treatment of AKI. Therefore, more studies on senescence, reprogramming, and AKI should be conducted to explore more senolytic therapies.
